# HBK-10, A Compound with α_1_-Adrenolytic Properties, Showed Antiarrhythmic and Hypotensive Effects in Rats

**DOI:** 10.3390/ph15101256

**Published:** 2022-10-12

**Authors:** Klaudia Lustyk, Kinga Sałaciak, Agata Siwek, Barbara Filipek, Jacek Sapa, Henryk Marona, Dorota Żelaszczyk, Karolina Pytka

**Affiliations:** 1 Department of Pharmacodynamics, Faculty of Pharmacy, Jagiellonian University Medical College, Medyczna 9, 30-688 Krakow, Poland; 2 Department of Pharmacobiology, Faculty of Pharmacy, Jagiellonian University Medical College, Medyczna 9, 30-688 Krakow, Poland; 3 Department of Bioorganic Chemistry, Chair of Organic Chemistry, Faculty of Pharmacy, Jagiellonian University Medical College, Medyczna 9, 30-688 Krakow, Poland

**Keywords:** α_1_-adrenoceptor, 2-methoxyphenylpiperazine, arrhythmia, hypertension, adrenaline-induced arrhythmia

## Abstract

Arrhythmia, an irregular heartbeat, might be a life-threatening condition but also a risk factor for stroke or worsen the prognosis after myocardial infarction. The limited efficacy and proarrhythmic potential of the available drugs require searching for new, more effective, and safer pharmacotherapies. Studies indicate that the blockade of α_1_-adrenoceptors could be effective in treating heart rhythm abnormalities. In this study, we aimed to assess the antiarrhythmic and hypotensive potential of HBK-10, a novel 2-methoxyphenylpiperazine derivative, as well as its binding to the selected adrenergic receptors. Radioligand binding studies demonstrated that HBK-10 showed a high affinity for α_1_ but not for α_2_ or β_1_ receptors. Next, we evaluated the ability of HBK-10 to protect against an adrenaline-induced arrhythmia in rats. The compound showed potent prophylactic antiarrhythmic properties in this arrhythmia model. Notably, the compound did not show proarrhythmic potential in normotensive rats since it did not influence the ECG parameters at antiarrhythmic doses. Finally, the compound showed hypotensive properties in rats, which were not observed after coadministration with adrenaline, noradrenaline, or methoxamine, which suggests α_1_-adrenolytic properties of HBK-10. Our results confirm that compounds with a 2-methoxyphenylpiperazine group show a high affinity for α_1_-adrenoceptors and a significant antiarrhythmic effect. Given the promising results of our study, further evaluation of HBK-10 is necessary to unravel the mechanisms behind its pharmacological effects and evaluate the safety profile.

## 1. Introduction

According to the World Health Organization, cardiovascular diseases are the leading cause of death globally [1]. Approximately 85% of deaths are due to myocardial infarction or stroke [1]. Cardiac-related risk factors, such as heart rhythm disturbances, are often involved in the mechanisms of these two conditions [2]. For example, atrial fibrillation increases fivefold the risk for acute ischemic stroke [3,4]. On the other hand, 90% of patients with acute myocardial infarction have some cardiac rhythm abnormalities [5]. Thus, restoring normal rhythm in these patients is of utmost importance. Antiarrhythmic drugs either block β_1_ receptors or influence sodium, potassium, or calcium channels [6]. Unfortunately, the above pharmacotherapy is often ineffective in treating heart rhythm abnormalities [7,8]. Furthermore, antiarrhythmic drugs have proarrhythmic potential, which may increase the risk of a cardiac event [9,10,11]. Thus, searching for novel compounds with different mechanisms of action devoid of proarrhythmic potential is necessary.

Several studies have proved that α_1_-adrenoceptors might play a vital role in arrhythmias [12]. Experiments have shown that during both early ischemia and reperfusion, there is an enhanced responsivity to α-adrenergic stimulation [13,14]. The increased α-adrenergic responsivity may be due to an increase in α_1_ receptors in the ischemic myocardium originating from a site distinct from the intracellular site for trafficking of β-adrenergic receptors, possibly within or near the sarcolemma [12]. Interestingly, an α_1_ receptor blockade induced a potent antiarrhythmic effect in several species [13,14,15,16,17,18,19,20,21]. Overall, these findings indicate that targeting α_1_-adrenoceptors could be an effective strategy to reduce the incidence of sudden cardiac death due to heart rhythm abnormalities.

Studies demonstrated that compounds containing 2-metoxyphenylpiperazine group show a high affinity for α_1_-adrenoceptors and often antiarrhythmic activity [22,23,24]. Thus, we have selected an aroxyalkyl derivative of 2-metoxyphenylpiperazine, compound HBK-10 (*N*-(3-(2,6-dimethylphenoxy)propyl)-1-(4-(2-methoxyphenyl)piperazin-1-yl)butan-2-amine dihydrochloride), which in our previous studies showed antidepressant-like effects in mice [25]. Since HBK-10 contains 2-methoxyphenylpiperazine group, we hypothesized that it could bind potently with α_1_-adrenoceptors and consequently show cardiovascular effects. Therefore, our study aimed to assess its affinity for the selected adrenergic receptors and antiarrhythmic and hypotensive potential in rats.

## 2. Results

### 2.1. HBK-10 Showed a High Affinity for α_1_-Adrenoceptors

The studied compound possessed a high affinity for α_1_-adrenoceptors but not for α_2_ or β_1_ receptors (Table 1).

### 2.2. HBK-10 Showed a Prophylactic Antiarrhythmic Activity in the Adrenaline-Induced Arrhythmia

**Figure 1:**
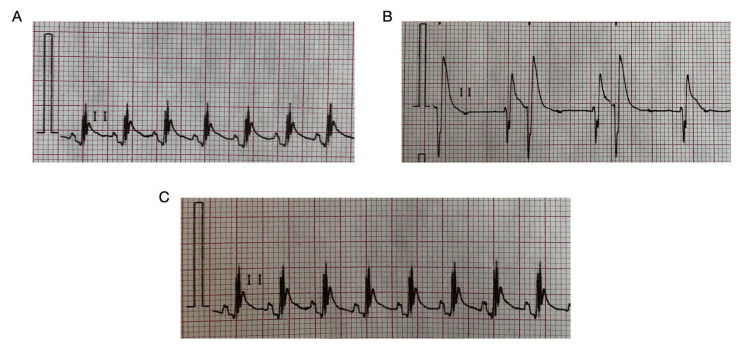
Representative ECG traces after treatment with adrenaline and/or HBK-10. (**A**) Normal reading (Control). (**B**) Arrhythmia control—adrenaline (20 μg/kg, *iv*). (**C**) Adrenaline-induced arrhythmia (20 μg/kg, *iv*) + HBK-10 (0.25 mg/kg, *ip* injection 45 min prior to adrenaline).

The administration of HBK-10 before the arrhythmogen decreased the number of extrasystoles caused by adrenaline injection (ED_50_ = 0.125 (0.061–0.256), Table 2, Figure 1).

### 2.3. HBK-10 Did Not Influence Negatively the Normal Electrocardiogram in Rats

HBK-10 at the dose 10 mg/kg had no effect on PR [F(9,45) = 1.749, ns], QRS [F(9,45) = 1.349, ns], QT_c_ [F(9,45) = 0.4078, ns] or heart rate [F(9,45) = 1.009, ns] (Table 3). However, when HBK-10 was administered at the dose 20 mg/kg, it did not affect the PR [F(9,45) = 2.076, ns] or QRS [F(9,45) = 1.377, ns], but it decreased QT_c_ by 9.6%, 9.8%, 10.5%, 11.4%, 9.6%, 8.3%, 12.8%, 15, 20, 30, 40, 50, 60, and 80 min post injection, respectively [F(9,45) = 4.341, *p* < 0.001], and heart rate by 12.3%, 19.3%, 21.3%, 22.3%, 23.7%, 24.6%, 24.5%, and 24.1% after 10, 15, 20, 30, 40, 50, 60, and 80 min from the compound’s administration, respectively [F(9,45) = 19.17, *p* < 0.0001] (Table 3).

### 2.4. HBK-10 Showed the Hypotensive Effect in Normotensive Rats

**Figure 2:**
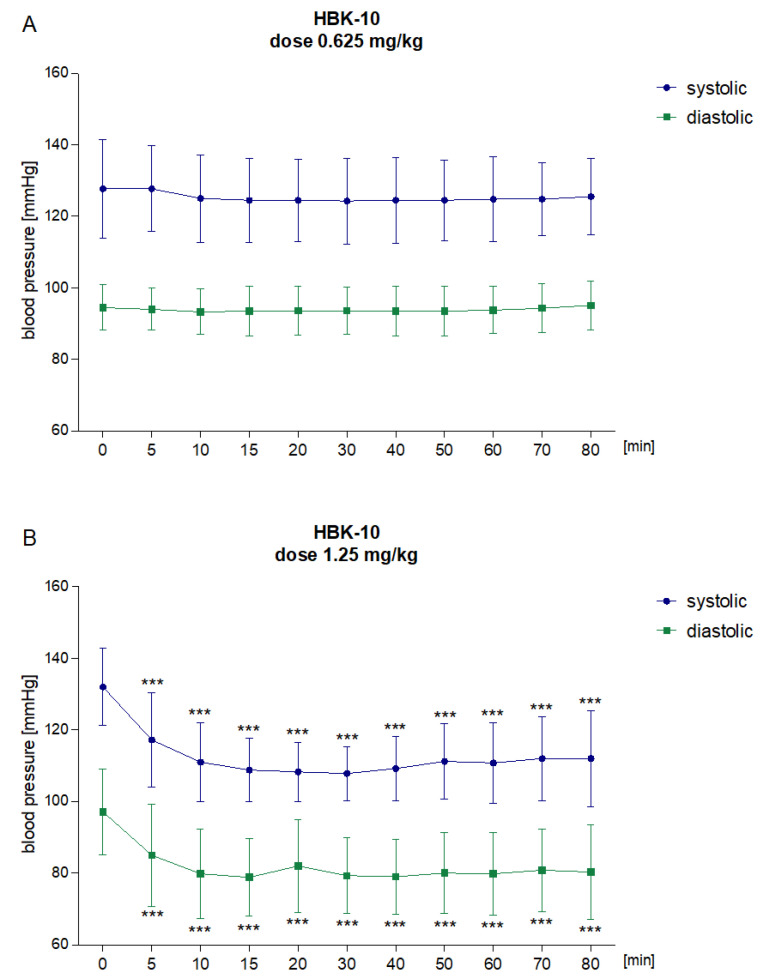
The effect of HBK-10, administered at doses 0.625 (Panel A) and 1.25 mg/kg (Panel B), on the systolic and diastolic blood pressure in normotensive rats. The tested compound was administered intraperitoneally (*ip*) and the blood pressure was measured 5, 10, 15, 20, 30, 40, 50, 60, 70, and 80 min after injection. The results are presented as means ± SD. Statistical analysis: one-way repeated measures ANOVA (Dunnet’s post hoc); *** *p* < 0.001 vs. blood pressure before the administration of the test compound (time point = 0 min); *n* = 6 rats.

HBK-10 did not affect the systolic [F(10,50) = 1.968, ns] or diastolic [F(10,50) = 1.712, ns] blood pressure at the dose 0.625 mg/kg (Figure 2A). However, when the test compound was given at the dose 1.25 mg/kg, 5 min after injection, it significantly reduced systolic blood pressure by 11.2–18.3% [F(10,50) = 23.3, *p* < 0.0001] and diastolic blood pressure by 12.5–18.3% [F(10,50) = 23.51, *p* < 0.0001] (Figure 2B). 

### 2.5. HBK-10 Attenuates Vasopressor Response after Adrenaline, Noradrenaline, and Methoxamine

**Figure 3:**
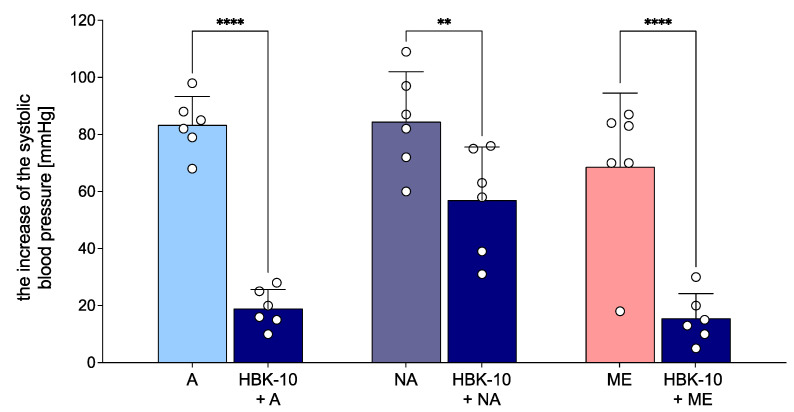
The effect of HBK-10 on adrenaline-, noradrenaline-, and methoxamine-induced pressor response. Pressor response to adrenaline (2 μg/kg), noradrenaline (2 μg/kg) and methoxamine (150 μg/kg) was estimated before and 5 min after the intravenous (*iv*) administration of HBK-10 at the dose of 1.25 mg/kg. The results are presented as means ± SD, circles represent individual data points. Statistical analysis: one-way repeated measures ANOVA (Bonferroni post hoc); ** *p* < 0.01, **** *p* < 0.0001; *n* = 6 rats. A—adrenaline, NA—noradrenaline, ME—methoxamine.

HBK-10 administered at the dose 1.25 mg/kg attenuated the adrenaline, noradrenaline, and methoxamine pressor response by 77.2%, 32.5%, and 77.4%, respectively [F(5,25) = 28.05, *p* < 0.0001] (Figure 3).

## 3. Discussion

In this study, we found that HBK-10, a novel 2-methoxyphenylpiperazine derivative, possessed a high affinity for α_1_-adrenoceptors. Furthermore, the tested compound elicited antiarrhythmic and hypotensive activity in normotensive rats, and its cardiovascular effects were most likely related to α_1_-adrenolytic properties. Notably, HBK-10 did not affect normal ECG in rodents. 

Numerous studies on 2-methoxyphenylpiperazine derivatives indicate their various biological properties, including cardiovascular effects, such as antiarrhythmic or antihypertensive [22,23,24]. Moreover, the compounds containing the 2-methoxyphenylpiperazine group often show a high affinity for the α_1_ receptors [28]. The α_1_-adrenoceptor, in turn, might play a vital role not only in the regulation of blood pressure, but also in antiarrhythmic effect [29,30]. Thus, we selected a novel 2-methoxyphenylpiperazine derivative—HBK-10—to determine its binding with the α_1_ receptor and the potential antiarrhythmic effect, as well as the influence on blood pressure. The compound in our previous studies demonstrated antagonistic properties at 5-HT_1A_ and D_2_ receptors and the antidepressant-like effect in mice [25].

First, we investigated the compound’s affinity towards adrenergic receptors, i.e., α_1_-, α_2_-, and β_1_-adrenoceptors, using radioligand binding assays. Our studies demonstrated that HBK-10 showed a high affinity for α_1_-adrenergic receptors, more robust than that of the reference compound, phentolamine. Compared with the α_1_ receptor, its affinities towards α_2_-, and β_1_-adrenoceptors were over 600- or 1000-fold lower, respectively. The radioligand binding studies suggest that among the adrenergic receptors, HBK-10 shows selectivity for α_1_-adrenoceptor. Furthermore, our studies confirmed that compounds containing the 2-metoxyphenylpiperazine group show a high affinity for α_1_ receptors. Our results align with our previous experiments [31] as well as other studies on 2-methoxyphenylpiperazine derivatives [28].

Since studies demonstrated a possible role of α_1_-adrenoceptors in the treatment of arrhythmia [23], in the following step, we investigated the potential antiarrhythmic activity of HBK-10 in the model of adrenaline-induced arrhythmia. In this model, adrenaline was used as an arrhythmogen, leading to arrhythmias manifested by extrasystoles, conduction blocks, bradycardia, and animal mortality. The tested compound showed prophylactic antiarrhythmic activity, reducing the number of post-adrenaline extrasystoles. It is worth highlighting that the calculated ED_50_ value for HBK-10 administered intraperitoneally was almost 3-fold lower than for carvedilol administered intravenously (ED_50_ = 0.36 mg/kg) [24]. Our results are even more promising given that compared with *iv* injection, after *ip* administration, a drug is subject to a hepatic first-pass metabolism, which decreases its systemic exposure. Moreover, as carvedilol is a potent β_1_- and α_1_-adrenoceptors blocker [32] and HBK-10 targets only α_1_-type adrenergic receptors, our results suggest that the α_1_-adrenoceptors might play a more significant role in the adrenaline-induced arrhythmia model than the β_1_-adrenoceptors. Since no fully effective antiarrhythmic drug is on the market, our results encourage further studies on HBK-10 as a model structure for synthesizing more effective drugs to treat heart rhythm disturbances.

Unfortunately, the available antiarrhythmic drugs show proarrhythmic potential that can manifest by the occurrence of *torsades de pointes*, an increased number of premature heartbeats, or reentry [33,34,35]. In particular, elderly patients with impaired heart muscle contractility and patients with electrolyte disturbances are susceptible to arrhythmogenic effects of antiarrhythmic drugs [36,37]. Therefore, next, we evaluated the influence of HBK-10 on normal ECG in rats. Our experiments revealed that HBK-10 influenced normal ECG at the dose 160-fold higher than the median effective dose in the adrenaline-induced arrhythmia model. The tested compound shortened the calculated QTc interval and decreased heart rate. The QT interval reflects depolarization and repolarization phases in the myocardium and its shortening indicates that the studied derivative may, to some extent, disrupt the heart rhythm at higher doses. The negative chronotropic effect of HBK-10 may be associated with the blockade of α_1_-receptors. However, considering that the dose necessary to disturb normal ECG is 160-fold higher than the antiarrhythmic dose, we can conclude that the compound did not show proarrhythmic potential at pharmacologically active doses.

Since α_1_-adrenergic receptors regulate blood pressure and α_1_-adrenolytics are being used in the treatment of hypertension [38], we next evaluated the effect of HBK-10 on systolic and diastolic blood pressure after a single intraperitoneal administration in normotensive rats. Our experiment demonstrated that HBK-10 reduced blood pressure at the dose 10-fold higher than the median effective dose in the adrenaline-induced model. The results suggest that the antiarrhythmic effect of HBK-10 requires lower doses than the hypotensive effect. The fact that these effects can be separated is beneficial. Compounds with such properties could be used in a larger group of patients, i.e., in patients with heart rhythm irregularities with or without hypertension.

Radioligand binding studies indicated the strength of a compound’s affinity for a given receptor without specifying the type of interaction. To prove the agonistic or antagonistic effect toward α_1_-adrenoceptors, we conducted the experiment with catecholamines. The inhibition of the pressor response to epinephrine, norepinephrine, or methoxamine by the pretreatment with the studied compound at the lowest hypotensive dose indicates its α_1_-adrenolytic properties. HBK-10 decreased the effect induced by epinephrine, norepinephrine, and methoxamine, suggesting that its hypotensive effect is due to the blockade of α_1_-adrenoceptors. However, we need to emphasize that HBK-10 targets serotonin 5-HT_1A_ and dopamine D_2_ receptors, which also regulate blood pressure. Therefore, the observed overall effect on blood pressure might result from the interaction with several receptors.

Considering the results of our experiments, the hypotensive effect of HBK-10 depended on the blockade of α_1_-adrenoceptors. Several studies showed significant antiarrhythmic effects of α_1_-adrenolytics [23,39,40]. Since HBK-10 showed a high affinity for the α_1_-adrenoceptors and negligible affinity for the β_1_-adrenoceptors, we may suspect that the observed antiarrhythmic effect was due to the interaction with α_1_-adrenoceptors. Nevertheless, to prove our hypothesis, we need further studies, as HBK-10 is a multimodal compound, and the observed antiarrhythmic effect might also be associated with the interaction with other biological targets.

Limitations to our study include assessing the antiarrhythmic and hypotensive activity of HBK-10 only after an acute administration. The repeated administration would verify the tested compound’s long-lasting effects on preventing attacks of arrhythmia, as well as increases in blood pressure in animal models. Moreover, to unravel the full antiarrhythmic potential of HBK-10, its activity in other arrhythmia models needs further investigation. Similarly, to understand the mechanism of action of HBK-10, electrophysiological studies should be performed and more potential molecular targets (e.g., ion channels) should be investigated in future studies.

## 4. Materials and Methods

### 4.1. Drugs

The studied compound, *N*-(3-(2,6-dimethylphenoxy)propyl)-1-(4-(2-methoxyphenyl)piperazin-1-yl)butan-2-amine dihydrochloride (HBK-10), was synthesized in the Department of Bioorganic Chemistry, Chair of Organic Chemistry, Faculty of Pharmacy, Jagiellonian University Medical College [25]. The investigated derivative was dissolved in saline (Polpharma, Duchnice, Poland) and administered intravenously (*iv*) or intraperitoneally (*ip*). 

Chemicals used in radioligand studies, i.e., phentolamine (Sigma-Aldrich, Hamburg, Germany), propranolol (Sigma-Aldrich, Hamburg, Germany), and clonidine (Sigma-Aldrich, Hamburg, Germany), were dissolved in saline. Commercially available reagents such as adrenaline (Polfa S.A., Warsaw, Poland), noradrenaline (Polfa S.A., Warsaw, Poland), and methoxamine (Sigma-Aldrich, Germany) were dissolved in saline and administered intravenously (*iv*). Thiopental (Sandoz GmgH, Kundl, Austria) was dissolved also in saline and administered intraperitoneally (*ip*). Heparin (Polfa S.A., Warsaw, Poland) was used as an anticoagulant during experiments. The control groups received saline as a vehicle. 

### 4.2. Animals

The experiments were performed on male normotensive Wistar rats, weighing 200–250 g. Animals were kept in standard plastic cages (42.7 cm × 26.7 cm) in groups of 3, at constant room temperature (22 ± 2 °C), on a 12 h light/dark cycle with ad libitum access to food and water. Each experimental and control group consisted of six animals. All injections were administered in a volume of 1 mL/kg. Rats were used only once in each test and immediately after each experiment, animals were euthanized. 

### 4.3. Radioligand Binding Assay

The α_1_-, α_2_-, and β_1_-adrenoceptor radioligand binding assay was carried out on the rat cerebral cortex. [^3^H]-prazosin (19.5 Ci/mmol, α_1_-adrenoceptor), [^3^H]-clonidine (70.5 Ci/mmol, α_2_-adrenoceptor), and [^3^H]-CGP-12177 (48 Ci/mmol, β_1_-adrenergic receptor) were used as specific ligands. The brains were homogenized using ULTRA-TURRAX homogenizer in 10 mL of an ice-cold 50 mM Tris–HCl buffer (pH 7.6). Homogenates were centrifuged at 20,000× *g* for 20 min (0–4 °C). Next, the cell pellet was resuspended in the Tris–HCl buffer and centrifuged again. Radioligand binding assays were performed in plates (MultiScreen/Millipore). The final incubation mixture (volume 300 μL) consisted of 240 μL of the tissue suspension, 30 μL of radioligand solution, and 30 μL of the buffer containing 7–8 concentrations of the tested compound. In order to measure the unspecific binding, 10 μM phentolamine (for [^3^H]-prazosin) or 10 μM clonidine (for [^3^H]-clonidine) or 1 μM of propranolol (in the case of [^3^H]-CGP-12177) were used. The incubation was terminated by rapid filtration through Whatman GF/C filters using a vacuum manifold (Millipore). The filters were then washed twice with the assay buffer and placed in scintillation vials with a liquid scintillation cocktail. Radioactivity was measured in a WALLAC 1409 DSA liquid scintillation counter (Perkin Elmer, Waltham, MA, USA). All the assays were made in duplicates and the inhibitory constants (Ki) were estimated.

### 4.4. Prophylactic Antiarrhythmic Activity in Adrenaline-Induced Arrhythmia

The experiment was carried out according to the method described by Szekeres and Papp [41]. Normotensive rats were anesthetized with thiopental (75 mg/kg *ip*). Adrenaline was administered at the dose of 20 µg/kg to induce heart rhythm disturbances. The studied compound was injected intraperitoneally 45 min before the arrhythmogen. The electrocardiogram (ECG) was recorded during the first 2 min and in the 5th, 10th, and 15th min after the adrenaline injection. The criterion of prophylactic antiarrhythmic activity was the lack of extrasystoles and inhibition of cardiac arrhythmia in comparison with the control group. The ED_50_ (a dose producing a 50% inhibition of ventricular contractions) was determined by computer log-probit analysis according to Litchfield and Wilcoxon [42]. 

### 4.5. Effect on a Normal Electrocardiogram in Rats 

To verify the influence of the tested compound on the normal electrocardiogram (ECG), the experiment was performed. Aspel ASCARD apparatus (standard II lead, with tape speed 50 mm/s and voltage calibration 1 mV = 1 cm) was used for ECG measurements. Normotensive rats were anesthetized with thiopental (75 mg/kg *ip*). The ECG was recorded prior and 5, 10, 15, 20, 30, 40, 50, 60, and 80 min after intraperitoneal administration of the tested compound. The influence on PQ, QT_c_ interval, QRS complex, and heart rate was evaluated. Bazzett’s formula, QT_c_ = QT/√RR, was used to calculate QT_c_ [27]. 

### 4.6. Influence on Blood Pressure in Normotensive Rats

Normotensive rats were anesthetized with thiopental (75 mg/kg *ip*). The right carotid artery was cannulated with a polyethylene tube filled with heparin solution to allow pressure measurements, using a Datamax apparatus (Columbus Instruments, Columbus, OH, USA) [41]. The tested compound was administered intraperitoneally after 15 min of stabilization period and its effect on blood pressure was measured. 

### 4.7. Influence on Blood Vasopressor Response in Rats

To establish agonistic or antagonistic toward α_1_-adrenergic receptors, we studied the influence of the studied compound on the pressor response to adrenaline (2 µg/kg), noradrenaline (2 µg/kg), and methoxamine (150 µg/kg). Normotensive rats were anesthetized with thiopental (75 mg/kg *ip*). The right carotid artery was cannulated with a polyethylene tube filled with heparin solution to allow pressure measurements, using a Datamax apparatus (Columbus Instruments, Columbus, OH, USA) [41]. The experiment was carried out after 15 min of the stabilization period. The pressor response to adrenaline, noradrenaline, and methoxamine was measured before (control) and 5 min after the administration of the tested compound. 

### 4.8. Statistical Analysis

The number of animals in groups was based on our previous studies [24]. Results are presented as either means ± SD or as a percentage of occurrence of specific events (extrasystoles). Where it was necessary, the normality of data sets and homogeneity of variance were determined using Shapiro–Wilk and Brown–Forsythe test, respectively. In our analysis, we used one-way repeated measures ANOVA, followed by Dunnet’s or Bonferroni post hoc. The reported *p* values of all ANOVAs used the Geisser–Greenhouse correction when the sphericity assumption was not met.

## 5. Conclusions

Our study confirmed that 2-methoxyphenylpiperazine derivatives show a high affinity for α_1_-adrenoceptors and potent antiarrhythmic effects in rats. We demonstrated that HBK-10, *N*-(3-(2,6-dimethylphenoxy)propyl)-1-(4-(2-methoxyphenyl)piperazin-1-yl)butan-2-amine dihydrochloride, ameliorated adrenaline-induced arrhythmia and decreased blood pressure in normotensive rats. The observed hypotensive effect was most likely due to its α_1_-adrenolytic properties. Importantly, HBK-10 did not show proarrhythmic potential. Given the promising results, further studies on the mechanisms behind HBK-10 cardiovascular effects and its safety profile should be continued.

## Figures and Tables

**Table 1:** The affinity of HBK-10 for adrenergic α_1_, α_2_, and β_1_ receptors.

Treatment	Adrenergic Receptors—*pK_i_*		
	α_1_^a,b^	α_2_^a,c^	β_1_^a,d^
HBK-10	8.55	5.72	5.52
Phentolamine	8.04	-	-
Clonidine	-	8.60	-
Propranolol	-	-	8.12

Data are represented as *pK_i_,* that is, −log*K_i_* and expressed as means from three independent experiments performed in duplicates. Inhibition constants (*K_i_*) were calculated according to the equation of Cheng and Prusoff [26]. ^a^ Radioligand binding was performed using rat cortex tissue. ^b^ The affinity values were determined using [^3^H]-prazosin. ^c^ The affinity values were determined using [^3^H]-clonidine. ^d^ The affinity values were determined using [^3^H]-CGP-12177.

**Table 2:** The effect of HBK-10 on extrasystole occurrence in the adrenaline-induced arrhythmia model in rats.

Treatment	Dose (mg/kg)	Extrasystoles (%)
Control	-	100
HBK-10	0.25	16.7
	0.125	50
	0.0625	83.3

Rats were anesthetized intraperitoneally *(ip)* with thiopental (75 mg/kg). The tested compound was administered *ip* 45 min before the experiment. The control group received no treatment except the administration of arrhythmogen. The observation was performed for 15 min after the intravenous (*iv)* injection of adrenaline (20 μg/kg), i.e., during the first 2 min, and in the 5, 10, and 15th min. Results are presented as a percentage of the occurrence of extrasystoles, *n* = 6 animals.

**Table 3:** The effect of HBK-10 on the normal ECG in rats.

Dose (mg/kg)	Parameters	Time of Observation (min)									
		0	5	10	15	20	30	40	50	60	80
10	PR	53.9 ± 2.8	53.0 ± 3.3	55.8 ± 2.0	54.9 ± 2.4	53.1 ± 4.4	52.2 ± 4.4	55.6 ± 2.6	53.6 ± 4.4	54.7 ± 3.1	54.9 ± 4.2
	QRS	22.6 ± 1.8	21.5 ± 1.4	23.2 ± 3.6	23.5 ± 2.6	23.6 ± 2.9	22.9 ± 1.6	24.5 ± 1.8	23.9 ± 2.5	23.2 ± 1.9	22.0 ± 1.4
	QTc	215.3 ± 19.3	213.2 ± 20.8	214.4 ± 20.0	212.4 ± 12.3	216.7 ± 13.8	213.7 ± 19.0	221.4 ± 16.9	218.8 ± 11.8	217.4 ± 22.0	218.3 ± 25.5
	Rate	357.1 ± 22.6	347.3 ± 25.2	347.9 ± 29.2	345.0 ± 32.7	343.4 ± 33.6	338.5 ± 26.3	337.5 ± 25.0	342.2 ± 25.8	344.5 ± 28.9	350.7 ± 28.1
20	PR	53.1 ± 2.8	52.4 ± 3.4	55.3 ± 1.7	54.2 ± 2.1	52.1 ± 4.2	51.3 ± 4.4	54.6 ± 2.4	52.5 ± 4.6	53.7 ± 3.0	53.8 ± 4.1
	QRS	21.8 ± 1.7	20.9 ± 1.2	22.7 ± 3.4	22.8 ± 2.2	22.7 ± 2.5	22.0 ± 1.5	23.6 ± 1.5	22.9 ± 2.3	22.1 ± 1.7	21.0 ± 1.3
	QTc	198.4 ± 6.1	190.9 ± 8.4	187.8 ± 14.7	179.4 ± 16.4 **	178.9 ± 21.9 **	177.6 ± 21.6l **	175.7 ± 20.5 ***	179.3 ± 20.7 **	182.0 ± 30.1 *	173.0 ± 6.6 ***
	Rate	317.4 ± 26.0	304.2 ± 32.0	278.4 ± 41.8 ***	256.2 ± 52.1 ****	249.9 ± 53.5 ****	246.5 ± 55.6 ****	242.1 ± 53.5 ***	239.2 ± 55.4 ***	239.5 ± 56.6 ***	241.0 ± 64.1 ***

Rats were anesthetized intraperitoneally *(ip)* with thiopental (75 mg/kg). The tested compound was administered *ip* and ECG was recorded for 80 min, i.e., in the 5, 10, 15, 20, 30, 40, 50, 60, and 80th min. Statistical analysis: one-way repeated measures ANOVA (Dunnett’s post hoc) ** *p* < 0.01, *** *p* < 0.001, **** *p* < 0.0001; *n* = 6 rats. QT_c_—calculated QT interval according to Bazzett’s formula: QT_c_ = QT/√RR [27].

## Data Availability

Data are contained within the article.
